# Fluorescence *In Situ* Hybridization for Diagnosis of Whipple’s Disease in Formalin-Fixed Paraffin-Embedded Tissue

**DOI:** 10.3389/fmed.2017.00087

**Published:** 2017-06-22

**Authors:** Peter Braubach, Torsten Lippmann, Didier Raoult, Jean-Christophe Lagier, Ioannis Anagnostopoulos, Steffen Zender, Florian Peter Länger, Hans-Heinrich Kreipe, Mark Philipp Kühnel, Danny Jonigk

**Affiliations:** ^1^Institute of Pathology, Hannover Medical School (MHH), Hanover, Germany; ^2^Aix Marseille University, CNRS, IRD, INSERM, AP-HM, URMITE, IHU Méditerranée Infection, Marseille, France; ^3^Institute of Pathology, Charité Medical University Berlin, Berlin, Germany; ^4^Department of Gastroenterology, Hepatology, and Endocrinology, Centre for Internal Medicine, Hannover Medical School (MHH), Hanover, Germany

**Keywords:** Whipple’s disease, *Tropheryma whipplei*, fluorescence *in situ* hybridization, immunohistochemistry, formalin-fixed paraffin-embedded tissue

## Abstract

Whipple’s disease (WD) is a rare chronic systemic infection with a wide range of clinical symptoms, routinely diagnosed in biopsies from the small intestine and other tissues by periodic acid–Schiff (PAS) diastase staining and immunohistological analysis with specific antibodies. The aim of our study was to improve the pathological diagnosis of WD. Therefore, we analyzed the potential of fluorescence *in situ* hybridization (FISH) for diagnosing WD, using a *Tropheryma (T.) whipplei*-specific probe. 19 formalin-fixed paraffin-embedded (FFPE) duodenal biopsy specimens of 12 patients with treated (6/12) and untreated (6/12) WD were retrospectively examined using PAS diastase staining, immunohistochemistry, and FISH. 20 biopsy specimens with normal intestinal mucosa, *Helicobacter pylori*, or mycobacterial infection, respectively, served as controls. We successfully detected *T. whipplei* in tissue biopsies with a sensitivity of 83% in untreated (5/6) and 40% in treated (4/10) cases of WD. In our study, we show that FISH-based diagnosis of individual vital *T. whipplei* in FFPE specimens is feasible and can be considered as ancillary diagnostic tool for the diagnosis of WD in FFPE material. We show that FISH not only detect active WD but also be helpful as an indicator for the efficiency of antibiotic treatment and for detection of recurrence of disease when the signal of PAS diastase and immunohistochemistry lags behind the recurrence of disease, especially if the clinical course of the patient and antimicrobial treatment is considered.

## Introduction

### Epidemiology, Clinical Presentation, and Therapy

Whipple’s disease (WD) is a rare disease with an approximate prevalence of less than three cases per million people investigated in the general European population (data from Italy) (1, 2). It is caused by chronic systemic infection with the gram-positive bacterium *Tropheryma whipplei* (*T. whipplei*), which belongs to the group of Actinobacteria, one of the largest subgroups in the Bacteria domain. Related infectious organisms include Mycobacterium and Corynebacterium species (3). *T. whipplei* are non-motile and rod-shaped, about 1.4 µm in length and surrounded by a trilaminar cell wall containing glycans at the external membrane (4). Humans represent the only known host of *T. whipplei* and, most commonly, middle-aged white men are affected (mean patient age is 55 years, 85% of patients are male). Patients with classic WD usually exhibit a wide range of non-specific gastrointestinal symptoms (e.g., diarrhea, abdominal pain, and malabsorption), ascites, weight loss, anemia, lymphadenopathy, and fever (1, 5, 6). WD is a multi-systemic disease with manifestation in various extra-intestinal organs including the central nervous system (CNS) (10–50% of WD patients), the heart (endocarditis), joints (isolated arthritis, spondylodiscitis), the eye (uveitis), and lung (pneumonia) (7).

Notably, patients can also be asymptomatic carriers of *T. whipplei*, with up to 11% of the general European population testing positive in feces (data from Switzerland) (8, 9). *T. whipplei* is endemic in sewer workers and hyper-endemic in rural Senegal, as well as in families of patients and carriers (10, 11). Exposure to contaminated soil has been described as a possible way of infection with *T. whipplei* (1, 10, 12, 13); however, the most common infection transmission route is *via* the fecal-oral route. Close contact to carriers (1, 10, 11, 14) poor hygiene and living conditions (14), and the absence of toilets (11) are considered risk factors for infection with *T. whipplei*.

Inherited or acquired immunodeficiency is suspected to be required for the development of classic WD (15–17). The characteristic duodenal histology, with marked infiltration of the intestinal mucosa by macrophages, which can engulf *T. whipplei* but are incompetent to fully degrade them, can be explained by the lack of excessive local inflammation and an alternation in the phagocytic cell activation toward the phenotype of M2 macrophages (18).

Until the introduction of antibiotic treatment in the 1950s, WD was considered fatal due to progressive cachexia or involvement of the CNS (1, 6, 18–20). Presently, WD is managed by an antibiotic regimen consisting of an initiation therapy with ceftriaxone followed by retention therapy with co-trimoxazole orally over the course of 1 year (1, 19, 20).

### Diagnostic Approaches

#### History of Diagnosis

George H. Whipple first described WD and suspected a bacterium as the causative agent in 1907. In 1949, intra-mucosal macrophages with a granular, periodic acid–Schiff (PAS) reaction positive cytoplasm in the small intestine were described, suggesting degraded bacteria as a cause of WD. The actual bacterium, *T. whipplei*, was first detected by electron microscopy (EM) in 1960 (21). In the 2000s, the first cultivation of *T. whipplei* was achieved by the centrifugation-shell-vial technique and a human fibroblast cell line (human erythroleukemia cell line) (22), and its whole genome was analyzed subsequently (3, 23).

Currently, a variety of techniques are applied for the diagnosis of WD, including conventional histopathology, immunohistochemistry, EM, PCR-based assays, and microbial culture.

#### Histopathologic Diagnosis

In most routine cases, duodenal biopsies represent the first step in diagnosing WD (24). The histological hallmark of WD is a mucosal infiltration of foamy macrophages containing large amounts of PAS positive, diastase-resistant particles in the lamina propria. Even if gastrointestinal symptoms are minimal or absent, biopsies of most patients show PAS positive macrophages in this location.

In a study of 48 cases of WD, von Herbay et al. (16) categorized four different phenotypes (types 1–4) of macrophages according to morphological characteristics, using PAS diastase staining (16). In WD, foamy PAS diastase positive macrophages are a distinct cytological feature in light and EM, which is not altered by routine processing of biopsies. Type 1 macrophages show a predominantly granular and intensively PAS positive cytoplasm, whereas in type 3 macrophages, the cytoplasm is described as diffusely granular with faint PAS positivity. Type 2 macrophages represent an intermediate morphology between types 1 and 3, with a grossly granular and intensively PAS positive cytoplasm with a diffuse or finely granular, faintly PAS positive background (Figure S1 in Supplementary Material). Type 4 macrophages show a predominantly foamy, only slightly PAS positive cytoplasm, or miss PAS positivity altogether (16).

In WD, progression and response to therapy can be roughly estimated by the morphological classification of the local macrophage subtypes outlined above (25). von Herbay et al. (16) showed that PAS positive type 1 macrophages predominate in patients before antibiotic treatment. During treatment, the number of type 2 macrophages increases and after about 1 year of therapy, type 3 macrophages dominate in the intestinal mucosa (16, 25). Consequently, PAS positive materials persist in macrophages, despite successful treatment and clinical cure (25). This incomplete elimination of *T. whipplei* in WD patients underpins the hypothesis put forward in several studies that a (local) immunodeficiency is required for the development of actual WD (18). It should be noted, however, that a negative PAS staining does not rule out WD.

In extra-intestinal disease, macrophages containing PAS positive, diastase-resistant inclusions can also be found in tissues and liquids such as liquor, the CNS, lymph nodes, synovial fluid, cardiac valves, or the bone marrow. Consequently, in cases with clinical suspicion of WD and negative duodenal biopsy, other anatomic sites should be investigated and potentially biopsied (7).

### Electron Microscopy

Using EM, *T. whipplei* was and can be successfully visualized in various stages of degradation in the extracellular spaces and in macrophages of WD patients, for example in duodenal biopsies. However, due to its time consuming analysis, EM is no longer recommended as a diagnostic technique for WD (16, 21, 26).

### Microbiological Culture

*Tropheryma whipplei* can be cultured by the centrifugation-shell vial technique and a human fibroblast cell line (human erythroleukemia cell line); however, this approach is a time consuming method due to the generation time of 18 days of *T. whipplei*. Microbial culture has successfully been applied as method for the detection of *T. whipplei* from synovial fluids and feces of patients with WD (3, 22, 27, 28).

### Immunohistochemical Staining

With the development of a *T. whipplei* specific polyclonal antibody, immunohistochemistry has become an important tool in the diagnostic algorithm of WD (29, 30). Immunohistochemistry represents a specific method for diagnosing WD even in patients with atypical manifestations and when PAS stains lacks specificity (16, 17, 24, 30).

Corresponding to PAS staining, anti-*T. whipplei* immunohistochemistry remains positive during the course of antibiotic treatment. The degraded remains of *T. whipplei* persist in the affected macrophages, and because the antibody links to epitopes in the wall of the bacteria, macrophages stain positive during and after treatment (1, 29, 30).

However, *T. whipplei* specific polyclonal antibody has to be produced in macrophages of rabbits and is not commercially available.

### Polymerase Chain Reaction

*Tropheryma whipplei*-specific quantitative polymerase chain reaction (qPCR) is a reliable and specific method for diagnosing infections with *T. whipplei*, if confirmed by sequencing of multiple *T. whipplei* target genes to avoid false positive results. A singular positive result from gastrointestinal tissue may be the result of a simple colonization with *T. whipplei*. An actual WD should be suspected if saliva and feces specimens are both tested positive *via* qPCR and clinical symptoms are also present (31, 32). qPCR has also been described as a supportive diagnostic tool for the analysis of synovial fluid in suspected seronegative rheumatic diseases (33).

PCR analysis from cerebrospinal fluid is highly recommended in all WD cases, because asymptomatic *T. whipplei* infection of the CNS has been described in up to 40% of all patients with gastrointestinal manifestation of WD and represents a complication with a high mortality (1, 7, 17).

### Bacterial Species-Specific Fluorescence *In Situ* Hybridization

Bacterial species have previously been detected *in situ* by hybridizing fluorescent oligonucleotide probes (of 15–30 base pairs in length) targeted against specific sequences in either the 16S or 23S ribosomal RNA (rRNA) of bacteria (34–38). Fluorescence *in situ* hybridization (FISH) can be applied on cytological smears of fluids and formalin-fixed paraffin-embedded (FFPE) specimens alike. rRNA is abundant (~10^5^ copies per cell) and evenly distributed in the cytoplasm of bacteria, resulting in a positive staining of the whole bacterial cell by the respective FISH probe (34–40). Preamplification of specific rRNA sequences is not a prerequisite for this approach. Stained specimens can be analyzed by standard epifluorescence microscopy (34–40). The FISH probes can be individual designed and labeled, but a large amount of probes for diverse bacteria is also commercially available so far.

Fredricks et al. (5) investigated the localization of *T. whipplei* using FISH and confocal laser scanning microscopy in six intestinal and two lymph node specimens with WD and could describe the extracellular distribution of viable bacteria (5). Geissdorfer and colleagues (15) analyzed blood culture-negative endocarditis in 255 patients using microbiological culture, PCR, FISH, conventional histopathology, and immunohistochemistry: 16 cases tested positive for *T. whipplei* by PCR, while only one was positive by FISH (15). Audoly et al. (41) analyzed the impact of deglycosylation of a supposed *T. whipplei* biofilm on the discrepancies between diagnostic results in WD by FISH and immunofluorescence and hypothesized that surface glycoconjugates have a protective role for *T. whipplei* and should therefore be removed to allow efficient bacterial detection (41).

These studies, which show that *T. whipplei* can in principle be successfully detected and visualized by FISH, lead us to the question of whether FISH-based diagnosis can be a reliable tool in routine FFPE samples in cases of suspected WD. To this end, we used a specific probe for *T. whipplei* and established a protocol for FFPE routine specimens in our lab.

## Materials and Methods

We identified 20 biopsies of confirmed WD from the archives of the Institute of Pathology, Hannover Medical School (MHH) and the Institute of Pathology, Charité Berlin. In these patients, the diagnosis of WD was based on conventional histopathologic criteria using the PAS stains and immunohistochemistry using the only non-commercially available and specific antibody described by Lepidi et al. (30). Clinical data included age, sex, clinical manifestations, antimicrobial therapy, and course of the disease. These 20 biopsies were taken from 13 patients and belonged to 17 distinct cases (samples taken at the same time were summarized as a case). One sample (patient no. 7) was not sufficiently large for workup and was not further analyzed in this study. Serial sections were stained with hematoxylin and eosin (H&E) and PAS diastase stains, immunohistochemistry with the specific polyclonal (rabbit) anti-*T. whipplei* antibody and a species-specific anti-*T. whipplei* FISH probe.

The age of patients ranged from 37 to 70 years (mean: 54 years) and the age of the *T. whipplei* negative control group ranged from 2 to 75 years (mean: 44 years). The male/female ratio was 5:1. Biopsies and surgical specimens came from the small intestine (*n* = 14), lymph nodes (*n* = 2), brain (*n* = 2), and the colon (*n* = 1). Previous antimicrobial therapy was reported in 6 patients and specimens from different time points before and after therapy where available from 3 patients (see Table 1).

**Table 1:** Summary of patient data (age, sex, previous specific therapy of WD, and origin of tissue sample) including (consecutive) patient- and case numbers as well as laboratory IDs (patient no., case no., lab ID).

Patient no.	Case no.	Lab ID	Sex	Age	Time between biopsies	(Previous) therapy of WD	Sampling location
1	1	WD1	Male	57	–	No	Small intestinal mucosa
2	2	WD2	Male	64	–	Yes	Small intestinal mucosa
3	3	WD3	Male	50	–	No	Lymph node
4	4	WD4	Male	65	–	No	Small intestinal mucosa
5	5	WD5	Male	70	–	Yes	Small intestinal mucosa
6	6	WD6	Male	51	–	Yes	Brain
6	6	WD7	Male	51	Concurrent	Yes	Brain
8	7	WD9	Male	68	–	No	Small intestinal mucosa
9	8	WD10	Male	50	–	Yes	Small intestinal mucosa
9	9	WD11	Male	51	7 months	Yes	Small intestinal mucosa
9	9	WD12	Male	51	7 months	Yes	Mucosal membrane of the colon
10	10	WD18	Male	43	–	Yes	Small intestinal mucosa
10	11	WD13	Male	44	5 months	Yes	Small intestinal mucosa
10	11	WD14	Male	44	5 months	Yes	Small intestinal mucosa
10	12	WD15	Male	47	3 years	Yes	Small intestinal mucosa
11	13	WD16	Female	51	1 year	Yes	Small intestinal mucosa
11	14	WD17	Female	50	–	Yes	Small intestinal mucosa
12	15	WD19	Female	37	–	No	Lymph node
13	16	WD20	Male	70	–	No	Small intestinal mucosa

*If two or more biopsies were available, or the timepoint of primary diagnosis of WD was known, the time interval between the biopsies (or between primary diagnosis and biopsy) is given. In one sample (patient no. 7), the remaining tissue did not suffice for workup in this study*.

Controls were arbitrarily selected: gastric biopsies with confirmed *Helicobacter pylori* infection (*n* = 7), duodenal biopsies with mycobacterial infection (*n* = 3), and duodenal biopsies with normal histology (*n* = 10); all of these samples were taken from the archives of the Institute of Pathology of MHH.

This study was approved by the local ethics committee of MHH (no. 3381-2016) and performed in accordance to the Helsinki declaration.

A paraffin block containing bacteria of *Mycobacterium bovis, Staphylococcus aureus, Escherichia coli*, and *Aspergillus fumigatus* (Cell Control Array Bacteria plus Fungi, Zytomed Systems, Berlin, Germany) served as additional control. All specimens had been formalin-fixed overnight and subsequently paraffin-embedded using established histopathological protocols. 4-µm thick tissue sections were stained with H&E and PAS diastase, respectively, following standard histopathological protocols.

### Immunohistochemical Analysis

Immunohistochemical analysis was performed as previously described (30). Briefly, 3-µm thick sections were fixed to Super Frost slides (Thermo Scientific, USA), deparaffinized in xylene (2× 10 min) and rehydrated with graded ethanol (100, 85, 70, 50, 20%, each for 2 min), ending in distilled water. The slides were then incubated in 3% hydrogen peroxide for 10 min and non-specific antigen binding sites were blocked by treating the slides with Reagent 1 (ZytoChemPlus Horse Radish Peroxidase (HRP) Polymer Kit, Zytomed Systems, Germany) for 5 min. Afterward, the slides were incubated with a primary antibody for 1 h in a humidified chamber at room temperature. The primary antibody against *T. whipplei*, as described in “Whipple’s disease: Immunospecific and Quantitative Immunohistochemical Study of Intestinal Biopsy Specimens” by Lepidi et al. (30) and kindly provided by Dr. Didier Raoult (30), was used at a dilution of 1:500. Unbound primary antibody was removed *via* rinsing with washing buffer for 5 min, before Reagent 2 (ZytoChemPlus HRP Polymer Kit, Zytomed Systems, Germany) was used for signal enhancement for 20 min. After rinsing with washing buffer, slides were incubated with the secondary antibody (ZytoChemPlus HRP Polymer Kit, Zytomed Systems, Germany) for 30 min. The slides were rinsed again with washing buffer and the bound antibodies were detected with 3,3′-Diaminobenzidine (DAB, DAB Substrate Kit High Contrast, Zytomed Systems, Germany). Finally, sections were rinsed with distilled water for 15 min, cellular nuclei were stained for 2 min with haemalum, dehydrated in ascending Ethanol, and mounted with Eukitt media (Sigma-Aldrich, Germany).

### Fluorescence *In Situ* Hybridization

Sections of 3 µm thickness were fixed on Super Frost slides (Thermo Scientific, USA) and air dried overnight. Sections were deparaffinized (60 min at 60°C, 2× 10 min 100% Xylene, and 5 min 100% ethanol) and air dried. For dissolving components, FISH probes were preheated to hybridization temperature and 10 µl of the indocarbocyanine (Cy3) labeled *T. whipplei*-specific probe (Biovisible, Netherlands) mixture was applied on each section and covered airtight with a coverslip. After hybridization at 52°C in a humid chamber for 18 h, sections were uncovered and washed at hybridization temperature in a wash-buffer (MCW 5, Biovisible, Netherlands) for 10 min. Sections were then rinsed with distilled water, air dried, and mounted with 4′,6-Diamidin-2-phenylindol (DAPI) Dura Tec (Zytomed Systems, Germany) and a coverslip.

The sequence of the *T. whipplei*-specific probe (TAT TGC AAC CCT CTG TAC CA) had previously been described by Geissdorfer et al. in Ref. (15). Compared to all 16S rRNA entries in the blast NCBI (National Centre for Biotechnology Information), the *T. whipplei*-specific probe showed 100% coverage to *T. whipplei* with a five nucleotide mismatch to the next similar organisms (*Rhodanobacter* strains, *Dyella* strains, *Dokdonella* strains, *Tahibacter* strains, and *Frateuria* strains) (42, 43).

### Fluorescence *In Situ* Hybridization Combined with Immunofluorescence

In order to correlate conventional immunohistochemical analysis with FISH analysis, a combined immunofluorescence FISH analysis was performed.

First, FISH staining was carried out following our protocol described above. For the concomitant immunofluorescence staining, the primary antibody against *T. whipplei* was applied after rehydration with graded ethanol (100, 85, 70, 50, 20%, each for 2 min) finishing in distilled water, then incubating with 3% hydrogen peroxide for 10 min, and with Reagent 1 (ZytoChemPlus HRP Polymer Kit, Zytomed Systems, Germany) for 5 min. Subsequently, sections were washed and incubated with a cyanine (Cy2)-labeled secondary antibody (ImmunoResearch Laboratories Inc., USA) for 30 min. After washing, sections were air dried and mounted with DAPI Dura Tec (Zytomed Systems, Germany) and a coverslip.

### Analysis, Visualization, and Image Processing

Periodic acid–Schiff diastase staining was graded qualitatively, based on the morphological classification system described by von Herbay (16) and colleagues and semi quantitatively as described by Baisden et al. (16, 29).

The fluorescence slides were analyzed using an Olympus BX 51 epifluorescence microscope equipped with filters sensitive for DAPI (Excitation peak: 358 nm, DNA-emission peak: 461 nm and RNA-emission peak: 500 nm), Cy3 (Excitation peak: 550 nm, Emission peak: 570 nm), and Cy2 (Excitation peak: 492 nm, Emission peak: 510 nm) (Olympus corporation, Japan).

Photos were taken with a digital microscope (Keyence BZ9000 E, Osaka, Japan). Representative microphotographs were taken with filters for the detection of Cy3 (OP-66838 BZ TRITC Excitation wavelength: 540/25 Dichroic mirror wavelength: 565 Absorption wavelength: 605/55), Cy2 (OP-66836 BZ filter GFP-BP Excitation wavelength 470/40 Dichroic mirror wavelength: 495 Absorption wavelength: 535/50), and DAPI (OP-66834 BZ filter DAPI-BP Excitation wavelength 360/40 Dichroic mirror wavelength: 400 Absorption wavelength 460/50). Microphotographs were processed using the GNU Image Manipulation Program (GIMP, Version 2.8) on an IBM compatible PC running Windows 10.

## Results

### Routine Microscopy

Specimens of all 16 cases were morphologically classified using the system proposed by von Herbay et al. (16) using PAS diastase staining (16). In six biopsies, macrophages predominantly showed granular intensive PAS positive cytoplasm (type 1), eight biopsies showed a grossly granular and intensive PAS positive cytoplasm and a diffusely granular, slightly PAS positive cytoplasm in the background (type 2), and in five biopsies macrophages had a diffuse or finely granular, scarcely PAS positive cytoplasm (type 3) (Table 2).

**Table 2:** Fluorescence *in situ* hybridization results compared to previous therapy and morphological classification of macrophages.

Case no.	(Previous) therapy	FISH	Classification of macrophages
1	No	Positive	3
3	No	Negative	2
4	No	Positive	2
7	No	Positive	1
15	No	Positive	1
16	No	Positive	1
14	Yes	Negative	2
2	Yes	Positive	3
5	Yes	Negative	3
6	Yes	Negative	1
8	Yes	Negative	2
9	Yes	Negative	2
10	Yes	Negative	1
11	Yes	Positive	2
12	Yes	Positive	3
13	Yes	Positive	3

*The fluorescence *in situ* hybridization results were described as “positive,” when one or more *Tropheryma whipplei* (*T. whipplei*) was found in the biopsy and “negative,” when *T. whipplei* was absent in the biopsy. Classification of macrophages: macrophages were classified with 1–3 regarding to the morphological characterization of periodic acid–Schiff diastase-positive macrophages described by von Herbay et al. (16). (Previous) therapy of Whipple’s disease (WD): “no” indicates that no specific WD therapy has started at time of biopsy, “yes” indicates that a specific antibiotic WD therapy has already been started at time of biopsy*.

### Immunohistochemical Analysis

**Figure 1:**
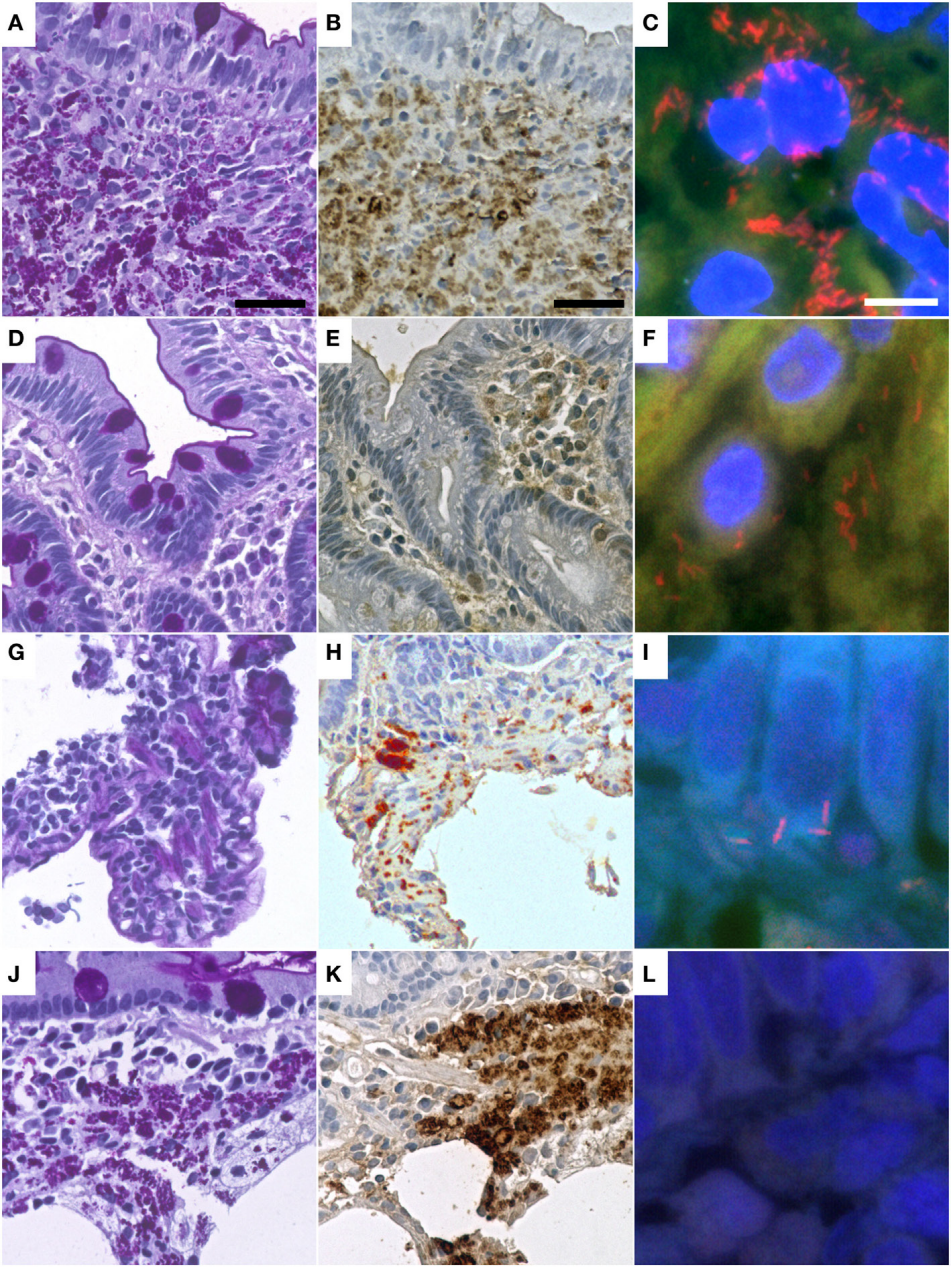
Representative cases of Whipple’s disease with periodic acid-Schiff diastase staining **(A,D,G,J)** show accumulated macrophages intensely positive in periodic acid–Schiff diastase staining and with positive reaction in immunohistochemistry with a specific anti-*Tropheryma whipplei* (*T. whipplei*) antibody **(B,E,H,K)**. Fluorescence *in situ* hybridization with a *T. whipplei* specific probe **(C,F,I,L)** shows differing numbers of intensely red-labeled bacteria ranging from dense aggregates **(C)** to sparse infiltrates of single bacteria **(F,I)**, while some cases did not show bacteria in FISH **(L)**. Presented cases are WD9 **(A–C)**, WD1 **(D–F)**, WD13 **(G–I)**, WD17 **(J–L)** (see also Table 2). The black bar is 30 µm. The white bar is 5 µm.

Specimens of all 16 cases with WD were positive for *T. whipplei* in immunohistochemical analysis, using the polyclonal (rabbit) anti-*T. whipplei* antibody described by Lepidi et al. (30) (Table 2). Staining was predominantly intracellular and congruent with foamy PAS diastase positive macrophages. In these cells, the pattern of antibody labeling was similar to the pattern of PAS diastase staining (Figure 1).

In all six patients with reported antimicrobial therapy, including three patients with longitudinal biopsies, immunohistochemical analysis for *T. whipplei* was positive. Congruent with reported results, the staining intensity declined after treatment, but nevertheless remained positive in all samples.

No specific positive signal for *T. whipplei* was detected in the control specimens.

### Fluorescence *In Situ* Hybridization

To detect *T. whipplei* in the FFPE specimens, FISH was used on routine tissue sections of duodenal biopsies, lymph nodes, and brain tissue. These specimens showed an excellent morphological quality.

In nine cases, *T. whipplei* could successfully be visualized using the species-specific FISH probe. In the seven remaining cases, it was not possible to detect *T. whipplei* organisms (Figure 1; Table 2). As expected, all negative controls showed no signals with the species-specific FISH probe. In all seven cases having a diagnosed *Helicobacter pylori* infection, rod-shaped bacteria were detectable in the mucus of the gastric mucosa in the DAPI channel. These were negative for the *T. whipplei*-specific FISH probe, however.

**Figure 2:**
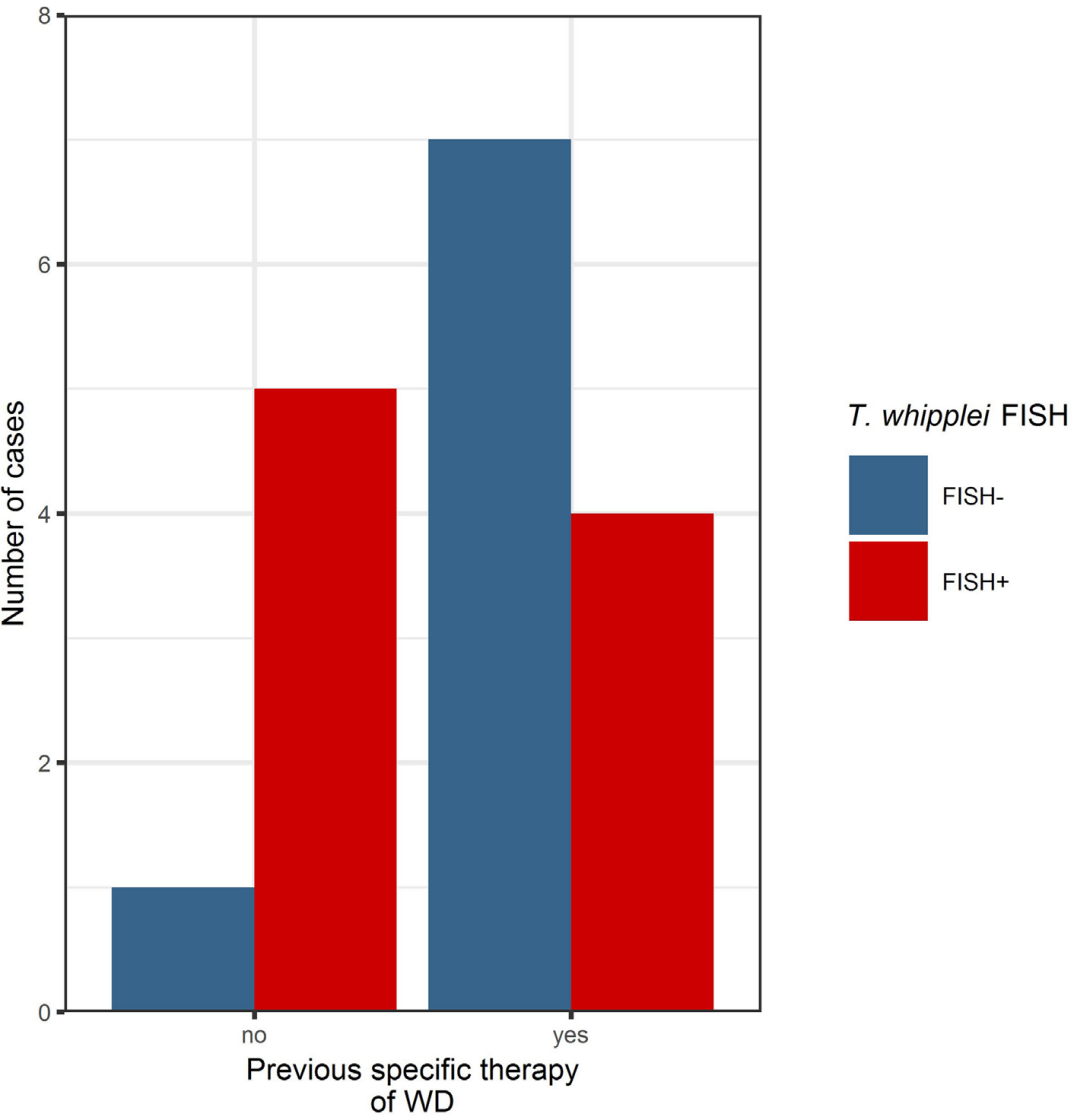
Before specific antibiotic therapy, the majority of cases investigated in this series were positive in the *Tropheryma whipplei* (*T. whipplei*) specific fluorescence *in situ* hybridization (FISH). After specific therapy, the majority of cases were FISH-negative, remaining positivity may indicate persistence or recurrence of disease (see also Table 2).

In three cases of WD with an abundance of PAS diastase positive macrophages with a type 1 classification (according to von Herbay et al.) and positive immunostaining, the FISH probe visualized a prominent number of *T. whipplei* bacteria. In these patients, a specific antimicrobial therapy before sampling had not been reported (Figure 2).

In two other cases without antimicrobial therapy and a type 2 and 3 classification [according to von Herbay et al. (16)] with a slightly positive immunolabeling signal, bacteria could easily be identified using FISH but were not distributed uniformly. In one case without reported antimicrobial therapy, no bacteria were detectable with FISH. The 3 FISH-positive patients who underwent a previous specific antibiotic treatment all showed no bacteria in the initial biopsy. Although, after a period of 2 years, the macrophages showed an intensive, granular PAS positive cytoplasm and a diffuse, more finely granular PAS positive cytoplasm in the background type 2 and 3 [according to von Herbay et al. (16)]; this pattern likely indicates an intestinal remission, and only/very few bacteria could be detected by FISH in the subsequent biopsy.

In the other cases, no bacteria could be detected *via* FISH, and most of these were classified as advanced WD with macrophages of type 2 or 3 [according to von Herbay et al. (16)].

**Figure 3:**
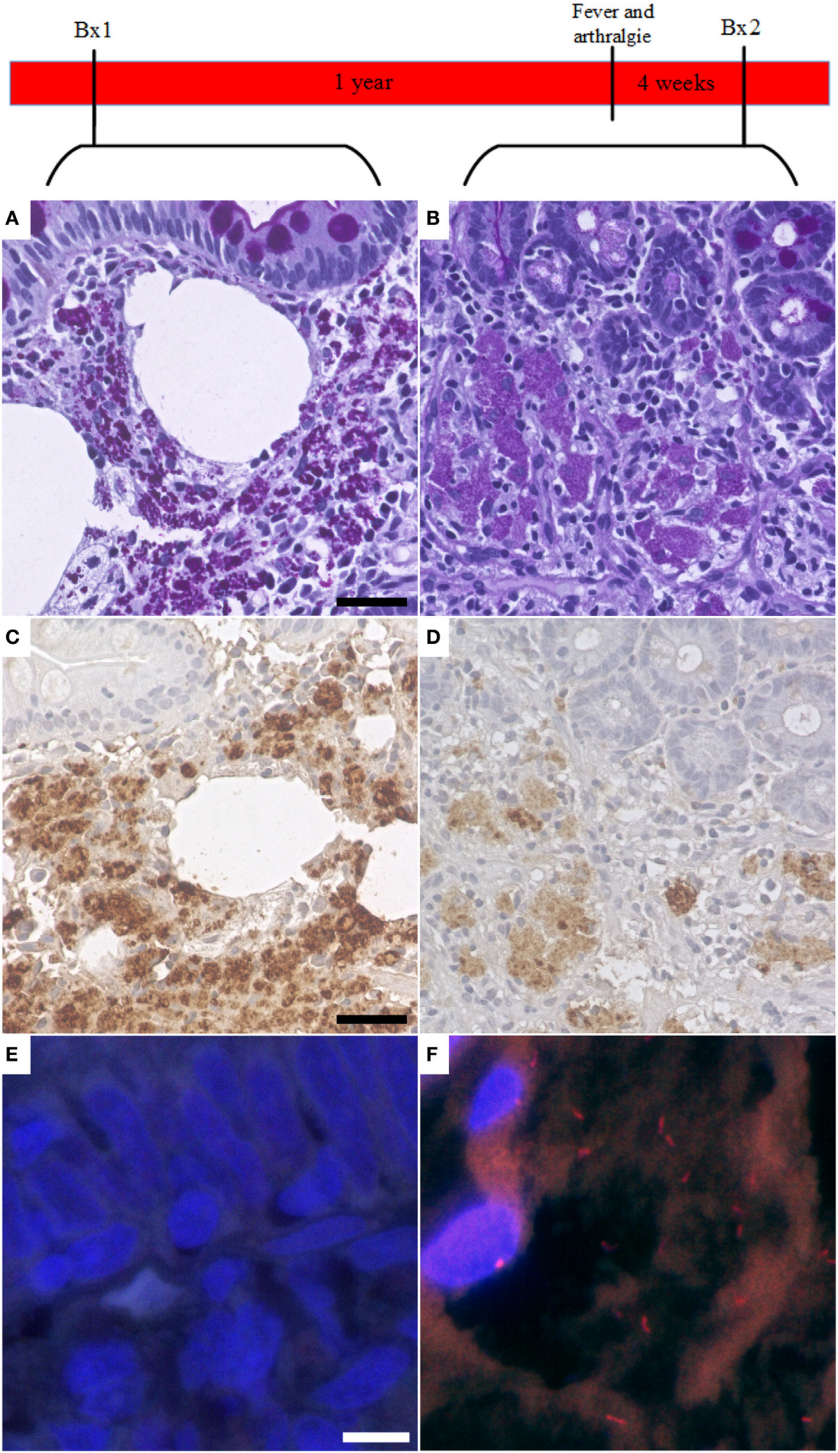
Fluorescent *in situ* hybridization (FISH) of *Tropheryma whipplei* (*T. whipplei*) may be a marker of disease recurrence. In one case, the first available biopsy (Bx1, taken after specific therapy) showed PAS diastase positive macrophages in the intestinal mucosa **(A)** and was positive in immunohistochemistry with the specific *T. whipplei* antibody **(C)**. However, it was negative in FISH **(E)**. One year after the biopsy, the patient presented with fever and arthralgia. A second biopsy (Bx2) was taken 4 weeks after onset of symptoms. This biopsy showed a reduction in PAS diastase positivity **(B)** and intensity of the specific immunohistochemistry **(D)** consistent with an effective therapy; however, in this biopsy, vital *T. whipplei* organisms could be detected in FISH **(F)**.

*Tropheryma whipplei* could be detected with FISH in 4 out of 10 cases with a specific antibiotic treatment, possibly indicating persistence or reoccurrence of active WD (Figure 3).

### Combination of Fluorescence *In Situ* Hybridization and Immunofluorescence

**Figure 4:**
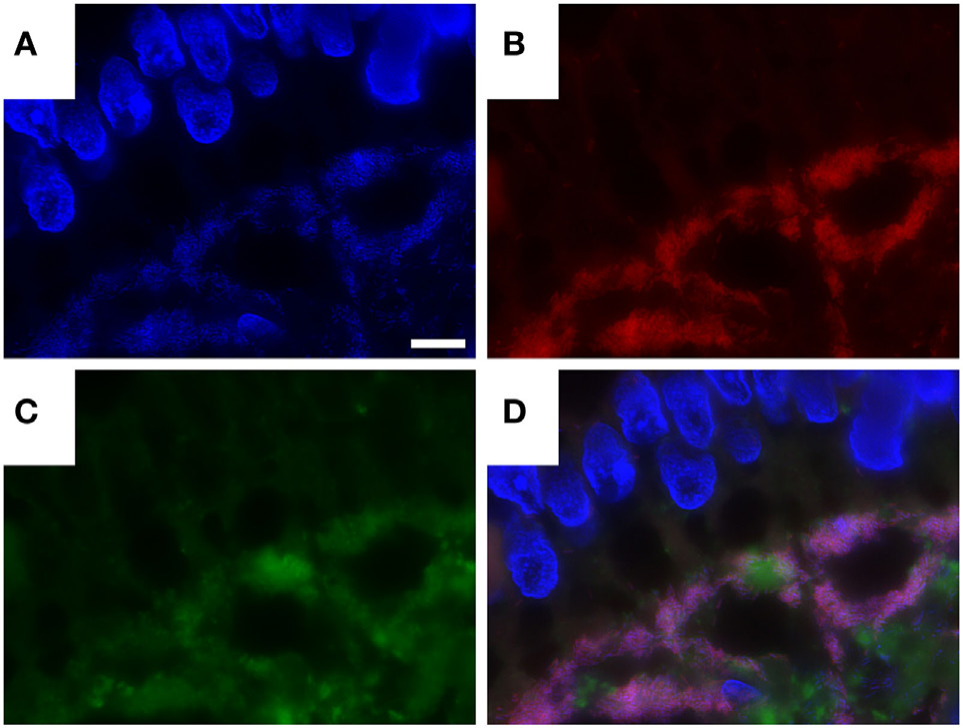
Combination of *Tropheryma whipplei* (*T. whipplei*) specific fluorescence *in situ* hybridization and fluorescence immunostaining with anti-*T. whipplei* antibody in an intestinal biopsy. 4′,6-Diamidin-2-phenylindol (DAPI) **(A)**, *T. whipplei*-specific probe (Cy3) **(B)**, anti- *T. whipplei* antibody with a fluorescence-labeled secondary antibody (Cy2) **(C)**, overlay of 4′,6-DAPI, *T. whipplei*-specific probe (Cy3), and anti- *T. whipplei* antibody with a fluorescence-labeled secondary antibody (Cy2) **(D)**. The white bar represents 5 µm.

Using a combination of *T. whipplei*-specific FISH and immunofluorescence labeling, we could show that FISH and the specific antibody consistently label very similar areas in the tissues examined. However, some immunohistochemical signals were also found separate from FISH-positive bacteria. In contrast to FISH, antibody labeling was consistently found co-localizing with the PAS diastase-positive macrophages at the intracellular, and also at the extracellular compartments (Figure 4).

## Discussion

In this study, we evaluated a species-specific FISH probe for the detection of *T. whipplei*, the bacterium causing WD, in FFPE tissue. We could detect *T. whipplei* in 56% (9/16) of the cases with clinically confirmed WD.

In the remaining cases, individual *T. whipplei* bacteria could not be observed. FISH negativity in cases positive by immunohistochemistry and PAS diastase may be attributed to one or more of the following scenarios: low ribosome content of bacteria (pointing toward an inactive state), a suspected *N*- and *O*-glycoconjugates containing biofilm surrounding *T. whipplei* inside macrophages (impermeable for the FISH probe), difficulties of probes to permeate cell walls or the inaccessibility of specific target sites due to tertiary structures or the effect of ribosomal proteins (4, 34, 41, 44).

Fluorescence *in situ* hybridization probes hybridize with bacteria, which contain high levels of rRNA, implying that hybridization depends on the metabolic activity of bacteria. A possible explanation for immunohistochemical positive and FISH-negative biopsies is the prior antibiotic treatment of WD. Due to the application of bacteriostatic and bactericidal antibiotics, such as ceftriaxone, bacteria are either arrested in their growth cycle or killed, both resulting in a decreased metabolic activity. This can result in an rRNA content well below the threshold required for detection by FISH leading to false negative results.

Considering the likely impact of prior antibiotic treatment on FISH results, we divided our study cohort into two groups, representing specimens from patients with antibiotic treatment and those without. *T. whipplei* bacteria were detectable by FISH in both samples from treated and untreated patients. In patients with no specific antibiotic therapy, 83% of all biopsies were positive in FISH analysis. In patients undergoing antibiotic therapy, 40% of cases were positive. Application of an antibiotic regimen is therefore a plausible explanation for negative FISH results; however, single bacteria could also be visualized in 40% of cases with antibiotic treatment. In all FISH-positive cases from patients undergoing antibiotic therapy, bacteria could be detected in the second biopsy with a time lapse of 5 months to 2 years after initiation of therapy. This may represent a reactivation, persistence, or reinfection after an initially sufficient treatment (41).

Another reason for the potentially low rRNA content of bacteria in FISH-negative cases may be the fact that most *T. whipplei* bacteria are phagocytized by macrophages in the lamina propria. These macrophages display a foamy and granular cytoplasm, which stains positive *via* PAS diastase, representing the phagocytized and degraded bacteria, which are also positive in specific immunohistochemistry.

The differential distribution of degraded and inactive bacteria inside vacuoles of macrophages and of metabolically active bacteria predominantly in the extracellular compartment has been described by Fredricks and colleagues (1, 5, 15, 29, 30, 41). These authors also showed that a *T. whipplei*-specific FISH probe no longer hybridized in WD cases treated with RNAse, which leads to the assumption that enzymatic degradation actually destroys the probe’s target. The distribution of *T. whipplei* antigens or dead bacteria inside macrophage vacuoles and metabolically active bacteria in the extracellular space was also confirmed by a study of Audoly et al. (41). This aspect is also supported by our findings in the combined *T. whipplei*-specific FISH and immunofluorescence labeling, where signals of antibody stain and FISH did not fully colocalize. In addition, Audoly et al. (41) detected a *N*- and *O*-glykosylation surrounding *T. whipplei* cell material in unknown state of degradation within the PAS diastase-positive macrophages, suspecting a biofilm hindering the oligonucleotide probe to permeate (41).

The permeability of the cellular wall of bacteria for the oligonucleotide probes (with a molecular weight of about 6,500 Da) in formalin-fixed specimen is not fully resolved; however, FISH analysis with oligonucleotide probes was successfully used in several previous studies including smears and tissue preparations from human and animals but also whole cell hybridization of bacteria (34, 35, 38, 45, 46). The *T. whipplei* species-specific probe was analyzed, was also successful used in three studies before (5, 15, 41). Differences in the three-dimensional structure of the ribosome or the effect of ribosomal protein interactions could be reasons for negative results (44).

### Perspectives

In order to verify our assumption that treated and inactive cases of WD are not detectable *via* FISH, a larger cohort of untreated cases of WD has to be investigated. Ideally, this would mean longitudinal studies of patients with WD before and after antibiotic treatment. By using multi-labeled FISH probes, it might be possible to increase signal intensity to allow detection of bacteria with a low content of rRNA and by this improving the overall sensitivity of the assay (47, 48).

Combination of immunohistological staining using species-specific antibodies and FISH probes could allow new insights into the cellular composition of ongoing infection and distribution of bacteria in their natural habitat (5, 41).

In our study, we could show in 12 patients from routine practice that FISH has the potential to diagnose active WD in FFPE specimens and enables the direct visualization and detection of individual vital bacteria in the infected tissue (5, 6, 34, 38, 49, 50). While sensitivity of FISH is not yet ideal, the commercially available FISH represents a valuable addition to the diagnostic kit and may be improved by the deglycolisation method proposed by Audoly et al. (41). FISH has value as an ancillary diagnostic tool, if used correctly. Our results show that FISH may be a helpful method to detect recurrence of disease after antibiotic treatment when the signal of PAS diastase and the specific immunohistochemistry lags behind the recurrence of disease, especially if the clinical course of the patient and antimicrobial treatment is considered.

Fluorescence *in situ* hybridization should not be used for a definite exclusion of WD but can visualize and resolve individual vital *T. whipplei* bacteria on formalin-fixed specimen of active WD. It therefore may be considered an indicator for the efficiency of antibiotic treatment or a potential indicator of a reactivation or reinfection in the course of WD treatment.

## Ethics Statement

This study was approved by the local ethics committee of MHH (no. 3381-2016) and performed in accordance to the Helsinki declaration.

## Author Contributions

PB conceived the research, analyzed the data, and wrote the paper. TL conceived the research, performed the experiments, analyzed data, and wrote the paper. DJ conceived the research and wrote the paper. DR, J-CL, and IA provided specimen and antibody and critically revised the work. MK, FL, SZ, and H-HK critically revised the work and conceived the research. TL and PB contributed equally and share first authorship.

## Conflict of Interest Statement

The authors declare that the research was conducted in the absence of any commercial or financial relationships that could be construed as a potential conflict of interest.

## Abbreviations

CNS, central nervous system; Cy2, cyanine; Cy3, indocarbocyanine; DAB, 3,3′-diaminobenzidine; DAPI, 4′,6-diamidin-2-phenylindol; EM, electron microscopy; FFPE, formalin-fixed paraffin-embedded; FISH, fluorescence *in situ* hybridization; H&E, hematoxylin and eosin; NCBI, national center for biotechnology information; PAS, periodic acid–Schiff reaction; qPCR, quantitative polymerase chain reaction; (r)RNA, (ribosomal) ribonucleic acid; *T. whipplei, Tropheryma whipplei*; WD, Whipple’s disease.
