# Supplementary material for: Fluorescence In Situ Hybridization for Diagnosis of Whipple’s Disease in Formalin-Fixed Paraffin-Embedded Tissue
Source: Front Med (Lausanne). 2017 Jun 22;4:87. doi: 10.3389/fmed.2017.00087 (PMC5479881; doi:10.3389/fmed.2017.00087)
Supplement: Supplementary file 1 [file Table_1.PDF]

Supplementary Table 1

Patient No.	Case No.	Sex	Age at time of Intervention	Time between biopsy	Lab ID	(Previous) Therapy of Whipple's disease	Biopsy specimen location	Semi-quantitative analysis PAS-diastase	Classification of macrophages	Semi-quantitative analysis IHC	FISH	Semi-quantitative analysis FISH
1	1	male	57		MW 1	no	small intestine mucosa	+	3	+	positive	++
2	2	male	64		MW 2	yes	small intestine mucosa	++	3	++	positive	++
3	3	male	50		MW 3	no	lymph node of the left Axilla	+	2	++	negative	n.a.
4	4	male	65		MW 4	no	small intestine mucosa	+	2	+	positive	++
5	5	male	70		MW 5	yes	small intestine mucosa	++	3	+	negative	n.a.
6	6	male	51		MW 6	yes	brain	++	1	++	negative	n.a.
6	6	male	51	concurrent	MW 7	yes	brain	++	1	++	negative	n.a.
7		male	61		MW 8	yes	small intestine mucosa	n.a.	n.a.	n.a.	n.a.	n.a.
8	7	male	68		MW 9	no	small intestine mucosa	+++	1	+++	positive	+++
9	8	male	50		MW 10	yes	small intestine mucosa	+	2	++	negative	n.a.
9	9	male	51	7 month	MW 11	yes	small intestine mucosa	+	2	++	negative	n.a.
9	9	male	51	7 month	MW 12	yes	mucous membrane of the colon	+	2	+	negative	n.a.
10	10	male	43		MW 18	yes	small intestine mucosa	+	1	+++	negative	n.a.
10	11	male	44	5 month	MW 13	yes	small intestine mucosa	+	2	++	negative	n.a.
10	11	male	44	5 month	MW 14	yes	small intestine mucosa	+	2	++	positive	+
10	12	male	47	3 years	MW 15	yes	small intestine mucosa	+	3	+	positive	+
11	13	female	51	1 year	MW 16	yes	small intestine mucosa	++	3	++	positive	+
11	14	female	50		MW 17	yes	small intestine mucosa	++	2	+++	negative	n.a.
12	15	female	37		MW 19	no	lymph node	++	1	+++	positive	+++
13	16	male	70		MW 20	no	small intestine mucosa	+++	1	+++	positive	+++

Summary of patient data (age, sex, previous specific therapy of Whipple’s disease and origin of tissue sample) including (consecutive) patient- and case numbers as well as laboratory IDs (Patient No., Case No., Lab ID) and results of PAS-diastase, immunohistochemistry using an anti-*Tropheryma whipplei* antibody and Fluorescence *in situ* hybridization using a *Tropheryma whipplei* species-specific probe. If two or more biopsies were available, or the timepoint of primary diagnosis of Whipple’s disease was known, the time interval between the biopsies (or between primary diagnosis and biopsy) is given. In one sample (Patient No. 7) the remaining tissue did not suffice for workup in this study. The fluorescence *in situ* hybridization results were described as "positive", when one or more *Tropheryma whipplei* was found in the biopsy and "negative", when *Tropheryma whipplei* was absent in the biopsy. Classification of macrophages: Macrophages were classified with 1-3 regarding to the morphological characterisation of PAS-diastase positive macrophages described by von Herbay *et al.* (1996). (Previous) therapy of Whipple’s disease: "no" indicates that no specific Whipple’s disease therapy has started at time of biopsy, "yes" indicates that a specific antibiotic Whipple’s disease therapy has already been started at time of biopsy. Semi-quantitative analysis of PAS-diastase, Immunohistochemistry and Fluorescence *in situ* hybridisation (FISH) were graded as described by Baisden *et al.* (2002).: + if the feature was lacking in most microscopic fields or regions of interest, ++ if the feature was easily identified but not present uniformly in most microscopic fields or regions of interest, or +++ if the feature was frequently and uniformly present in high abundance in most microscopic fields or regions of interest.
