# Supplementary material for: Fluorescence In Situ Hybridization for Diagnosis of Whipple’s Disease in Formalin-Fixed Paraffin-Embedded Tissue
Source: Front Med (Lausanne). 2017 Jun 22;4:87. doi: 10.3389/fmed.2017.00087 (PMC5479881; doi:10.3389/fmed.2017.00087)
Supplement: Supplementary file 2 [file Image_1.PDF]

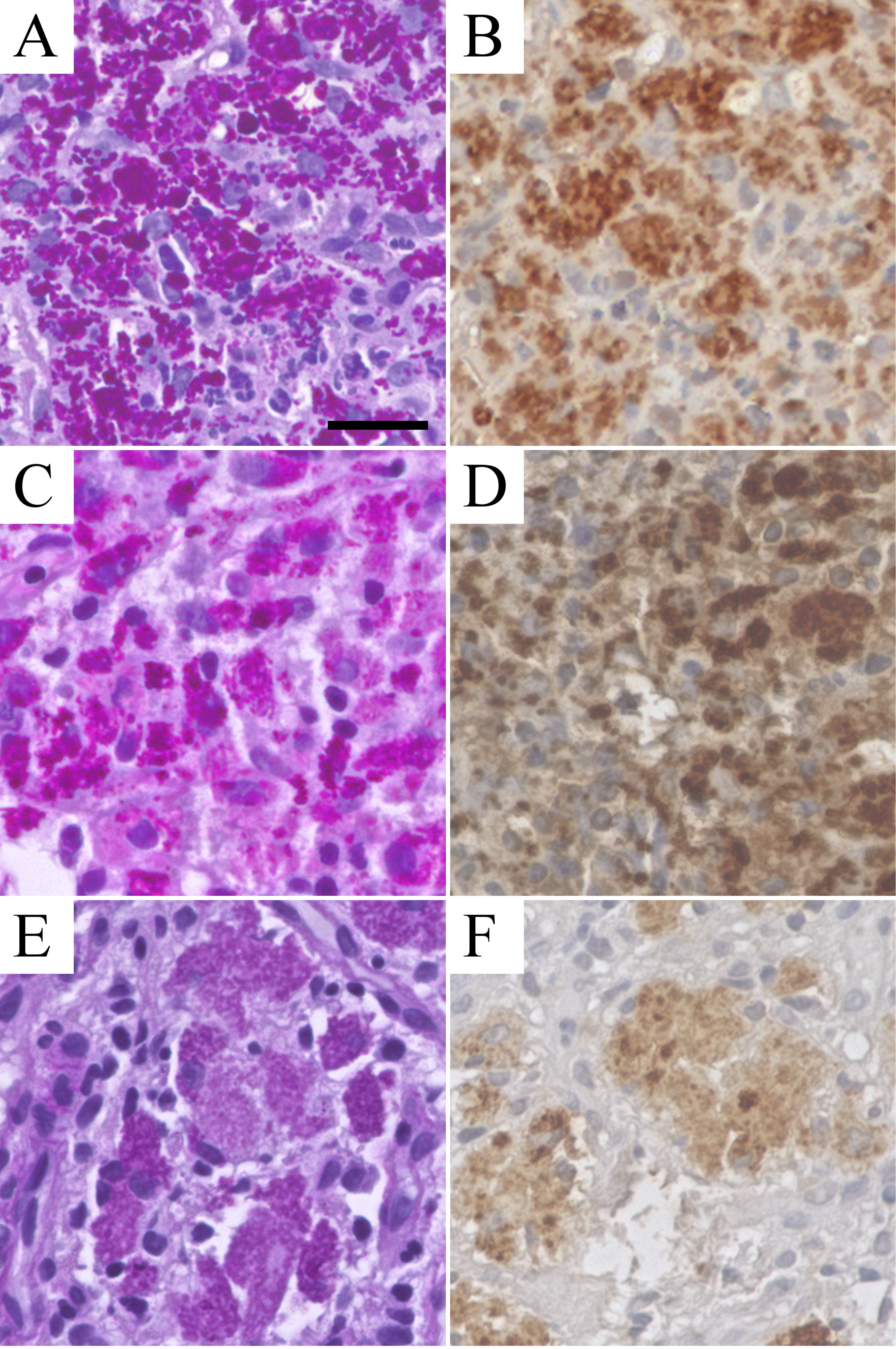

Supplementary Figure 1: Representative images of PAS-diastase (A, C, E) and immunohistochemistry using an anti-*Tropheryma whipplei* antibody (B, D, F). Macrophage types 1-3 according to von Herbay *et al.* (1996) show intensely PAS-diastase positive granular inclusions in type 1 (A) and diffuse positivity in type 3 (E). Type 2 consists of an intermediate phenotype with granular inclusions on a diffuse PAS-diastase positive background (C). Antibody labelling is similar to the pattern of PAS-diastase staining and the staining intensity declines after treatment, but nevertheless remaines positive (B, D, F). The black bar is 30 µm.
