# Supplementary material for: Fluorescence In Situ Hybridization for Diagnosis of Whipple’s Disease in Formalin-Fixed Paraffin-Embedded Tissue
Source: Front Med (Lausanne). 2017 Jun 22;4:87. doi: 10.3389/fmed.2017.00087 (PMC5479881; doi:10.3389/fmed.2017.00087)
Supplement: Supplementary file 3 [file Image_2.PDF]

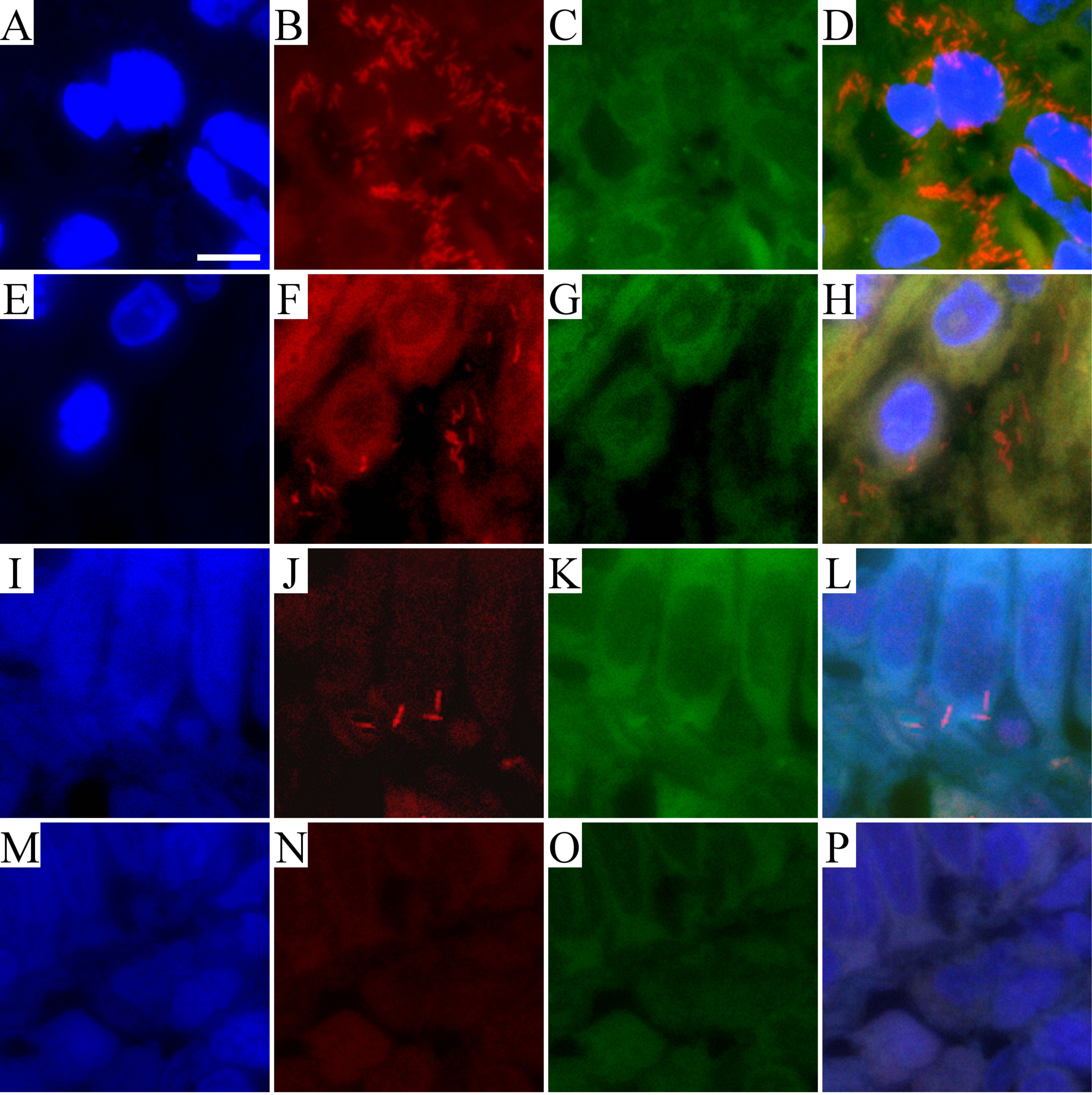


Supplementary Figure 2: Single channel images and merges of fluorescence in situ hybridization (FISH) shown in Figure 1. Blue is a nuclear counterstain with 4',6-Diamidin-2-phenylindol (DAPI) (A, E, I, M), red a Cy3 labelled *Tropheryma whipplei* specific FISH probe (B, F, J, N). Green is unspecific autofluorescence used for orientation (C, G, K, O). Last row presents the merges of the blue, red and green channel (D, H, L, P) also shown in Figure 1. Fluorescence *in situ* hybridization with a *Tropheryma whipplei* specific probe (B, F, J, N) shows differing numbers of intensely red labelled bacteria ranging from dense aggregates (B) to sparse infiltrates of single bacteria (F and J), while some cases did not show bacteria (N). Presented cases are WD9 (A, B, C, D), WD1 (E, F, G, H), WD13 (I, J, K, L), WD17 (M, N, O, P) (see also Supplementary Table 1). The white bar is 5 µm.
